# The Five Times Sit-to-Stand Test: safety and reliability with older intensive care unit patients at discharge

**DOI:** 10.5935/0103-507X.20190006

**Published:** 2019

**Authors:** Thiago Araújo de Melo, Antonio Carlos Magalhães Duarte, Thaysa Samanta Bezerra, Fabrícia França, Neila Silva Soares, Debora Brito

**Affiliations:** 1 Escola de Ciências da Saúde, Universidade Salvador - Salvador (BA), Brasil.; 2 Instituto Sócrates Guanaes - Rio de Janeiro (RJ), Brasil.; 3 Hospital Teresa de Lisieux - Salvador (BA), Brasil.; 4 Centro Universitário Estácio - Salvador (BA), Brasil.

**Keywords:** Hospitalization, Risk assessment, Accidental falls/prevention & control, Physical therapy modalities, Rehabilitation, Aged, Patient discharge, Intensive care units

## Abstract

**Objective:**

Assess the Five Times Sit-to-Stand Test safety and clinimetric properties in older patients hospitalized in an intensive care unit.

**Methods:**

Test safety was assessed according to the incidence of adverse events and through hemodynamic and respiratory data. Additionally, reliability properties were investigated using the intraclass correlation coefficients, standard error of measurement, standard error percentage change, Altman-Bland plot and a survival agreement plot.

**Results:**

The overall suitability of the Five Times Sit-to-Stand Test was found to be low, with 29.8% meeting the inclusion criteria. Only 44% of the hospitalized patients who met the inclusion criteria performed the test, with no need for discontinuation in any patient. Heart rate (79.7 ± 10.2bpm/86.6 ± 9.7bpm; p = 0.001) and systolic blood pressure (118 ± 21.4mmHg/129 ± 21.5mmHg; p = 0.031) were the only variables that presented a significant statistical increase, with no evidence of exacerbated response to the test. Additionally, no adverse events were reported from participating and both test-retest and interrater reliability were high (intraclass correlation coefficient ≥ 0.99).

**Conclusion:**

The Five Times Sit-to-Stand Test was proven to be safe and to have excellent reliability. Its clinical use, however, may be restricted to high-functioning older adults in hospital settings.

## INTRODUCTION

A sit-to-stand (STS) movement is considered a fundamental prerequisite for mobility and functional independence, since the movement is part of the various Activities of Daily Living (ADL).^(^^1^^)^ Whitney et al.,^(^^2^^)^ declared that significant functional limitations can occur when the ability to rise from a seat is impaired. Accordingly, the Five Times Sit-to-Stand Test (FTSST) is considered to be a useful, consistent and low-cost tool to assess sit-to-stand ability.^(^^3^^)^ The FTSST measures the time taken to stand five times from a sitting position as quickly as possible. Researchers have described its use as a measure of lower limb strength,^(^^4^^)^ balance control,^(^^5^^)^ fall risk^(^^6^^)^ and exercise capacity.^(^^7^^)^ Slower sit-to-stand times have been linked to an increased risk for recurrent falls,^(^^8^^)^ slow gait speed^(^^9^^)^ and deficits in other ADL living in community-dwelling older people.^(^^10^^)^ Furthermore, reduced exercise capacity and muscle force in the quadriceps have been found in *chronic obstructive pulmonary disease* patients who were unable to complete the FTSST.^(^^7^^)^

Although past research has produced normative values and data on predictive and concurrent validity and test reliability when used for patients with osteoarthritis,^(^^11^^)^ stroke,^(^^12,13^^)^ Parkinson's disease^(^^14^^)^ and back pain,^(^^15^^)^ as well as in older hospitalized subjects,^(^^16^^)^ an evaluation of the safety, reliability and validity of the FTSST in older subjects within the intensive care unit (ICU) setting has not yet been conducted. Moreover, few studies have assessed the hemodynamic, respiratory and metabolic functioning of patients performing the FTSST.

The FTSST has potential to be a valuable tool for clinicians seeking a bedside tool to assess sit-to-stand ability for older hospitalized patients. Such a tool might complement other resources when identifying walking capacity, fall risk, and functional independence recovery in this setting. Thus, this study had a twofold purpose: (a) to establish the safety of applying the FTSST on discharge from a critical care unit within a hospital setting, and (b) to determine FTSST test-retest and interrater reliability when used with older patients being discharged from a critical care hospital unit.

## METHODS

This cross-sectional study was conducted between July and December 2015 at *Hospital Teresa Lisieux*, in Salvador, Brazil. We recruited a convenience sample of 96 patients, of both genders, who were greater than or equal to age 60 years, discharged from the general ICU to the hospital ward at study entry. The study was approved by the Human Research Ethics Committee of the *Universidade Salvador* (UNIFACS) - Salvador, Brazil, under protocol no. 1.047.232, and all participants provided informed written consent to participate.

All participants were aged ≥ 60 years, were able to sit and stand without assistance, had clinical and hemodynamic stability - resting heart rate (HR) from 60 to 100bpm, had systolic blood pressure (SBP) < 160mmHg/diastolic < 100mmHg without using vasoactive drugs, had oxyhemoglobin saturation by pulse oximetry (SpO_2_) at rest ≥ 92% without oxygen supplementation and received medical authorization to perform the FTSST.

Patients were excluded if they had any (a) substantial pain that might affect participation, (b) cognitive impairment that led them to be unable to understand the test instructions, or (c) presence of fever, unstable angina, cardiac arrhythmias, cardiac resynchronization therapy, myocardial infarction within the last 2 months, unstable heart or respiratory disease. Clinical staff were instructed to discontinue the test if patients presented a SpO_2_ decrease below 92% without O_2_ support, respiratory rate (RR) > 22 incursions per minute (ipm), HR > 120 beats per minute (BPM), systolic blood pressure (SBP) > 180mmHg and/or diastolic blood pressure (DBP) > 100mmHg and subjective perception of exertion > 13 evaluated by the Borg Scale of perceived exertion.^(^^17^^)^

Two trained senior physiotherapists administered the test when patients were being discharged from the intensive care unit and transferred into the hospital ward. We assessed the safety of the FTSST as defined by an absence of exacerbated hemodynamic and respiratory response or adverse events such as dizziness, fall, dyspnea, chest pain or musculoskeletal pain.

A Dixtal^®^ multiparameter monitor (DX 2020, Philips, Brazil) was used to record demographic, clinical and vital signs data (HR, RR, SpO_2_, SBP, DBP and double product), as well as a printed version of the Borg Scale of perceived exertion in a traditional version.

### Five-Times Sit-to-Stand Test

The FTSST reproduces the act of sitting and standing for five repetitions as rapidly as possible.^(^^18^^)^ In this study, tests were administered three times on the same day, with a minimum interval of 30 minutes for recovery between each test run, yielding an average result between tests. An untimed trial was given with the objective of reducing the risk of learning effects for all patients.

The participants began the FTSST test sitting in an armless chair with a seat height of 43cm.^(^^19^^)^ Each participant was instructed to cross their arms over their chest and sit with their back against the upright backrest of the chair. The rater then demonstrated the correct technique to perform the test, including coming to a full stand, defined as an upright trunk with hips and knees extended. Timing began when the rater spoke the word "go" and stopped when the participant's buttocks reached the seat following the fifth stand.^(^^14^^)^ The raters required the patients to stand and sit five times "as quickly as possible" without physical assistance. Words of encouragement or body language to speed up were not used so that the patients could choose their own intensity of exercise. If the participants stopped during the test to rest, the raters would say, "You can stay seated if you would like and then continue standing whenever you feel able" and would not stop the timer. If the patient stopped before the 5 times and refused to continue, we registered the reason for stopping prematurely and excluded the participant's score from analysis.

The test performance was based on its duration; consequently, the shorter time taken by the patient, the better their functional condition would be. Vital signs were measured at the beginning and end of test, and the frequency of adverse events was recorded in a specific instrument.

### Statistical analysis

Statistical analyses were conducted with Statistical Package for Social Science (SPSS) software version 22.0 (IBM^®^ SPSS^®^, v. 22.0, Armonk, NY, USA) and Microsoft Excel 2011 (Microsoft Corporation, Redmond, WA, USA). The Kolmogorov-Smirnov test was used to evaluate the normality of the data. Student's t-test for paired samples was used to test whether hemodynamic and respiratory variable responses before and after the test were significantly different. We performed a one-way ANOVA to analyze possible differences between trials. We determined test-retest and interexaminer reliability by using the intraclass correlation coefficients (ICCs), Altman-Bland plot and survival agreement plot. Intraclass correlation coefficients were calculated using a two-way random effects consistency model.

Luiz et al.,^(^^20^^)^ 2003, proposed a new graphic approach to complement the Altman-Bland method for agreement analysis. It allows a simple interpretation of agreement that takes into account the limits of tolerance based on clinical importance. This new approach uses the Kaplan-Meier method, which is normally used for the analysis of survival data, and thus, the authors have named this approach a survival-agreement plot. The data analyzed were considered statistically significant when p ≤ 0.05.

## RESULTS

**Figure 1:**
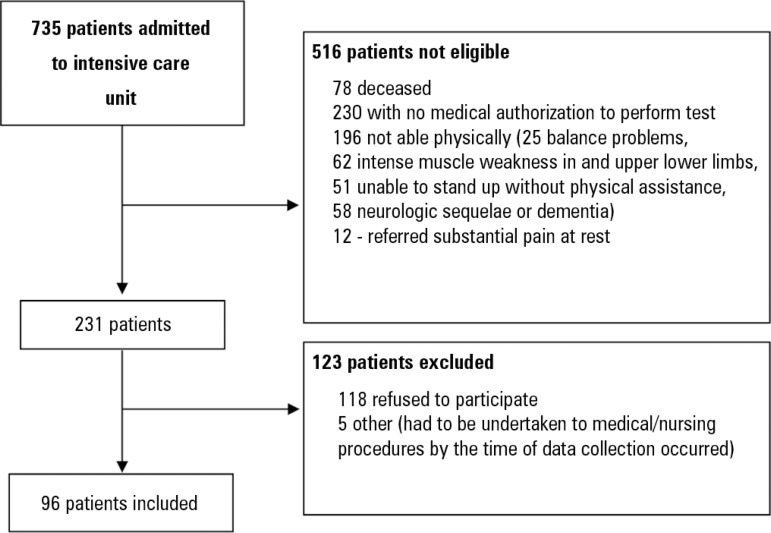
Flow chart of patients included in the study.

Figure 1 shows the flow chart of patients who met the inclusion/exclusion criteria for the study. As presented, 29.8% met inclusion/exclusion criteria, and, of these 43,8% gave informed consent to take the FTSST. All consenting patients (100%) were able to complete the test without incident.

Clinical and demographic data of the studied population are presented in table 1. The patients evaluated were 50% female, and the mean age was 61.70 ± 1.35 years. Regarding admitting diagnosis, 77% were admitted for clinical treatment and 23% for semi-urgent and elective surgical treatment - 57% cardiothoracic surgery, 23% abdominal surgery and 20% other (e.g., kidney biopsy, neurologic, etc). An average stay in the intensive care unit of 5.25 days ± 2.09 days was observed, with 56.2% of patients remaining for more than 3 days. Additionally, we observed that 16.7% used invasive ventilatory support, and only 8 patients had a medical diagnosis of sepsis (8.3%).

**Table 1:** Demographics and clinical characteristics

Clinical characteristics	
Age	61.70 ± 1.35
Sex	
Male	48 (50)
Female	48 (50)
Body mass index	24.61 ± 0.47
< 25kgm^2^ (eutrophic)	48 (50)
> 25kgm^2^ (overweight)	48 (50)
SAPS 3	32 ± 8.45
Admitting diagnosis	
Neurologic	6 (6.3)
Cardiologic	22 (22.9)
Surgery	22 (22.9)
Respiratory	12 (12.5)
Oncologic	6 (6.3)
Other (nephrological, gastrointestinal and hematologic disorders)	28 (29.2)
ICU length of stay (days)	5.25 ± 2.09
≤ 3	42 (43.7)
> 3	54 (56,2)
Use of invasive mechanical ventilation	
Yes	16 (16,7)
No	80 (83,3)
Use of mechanical ventilation support (days)	
> 3	12 (75)
≤ 3	4 (25)

SAPS 3 - Simplified Acute Physiology Score 3; ICU - intensive care unit. Values expressed as the mean ± standard deviation or N (%).

### Safety assessment

We performed 288 measurements of the FTSST, with no reports for discontinuation needed. Hemodynamic and respiratory variables were investigated to determine the safety of the test (Table 2).

**Table 2:** Hemodynamic and respiratory variables pre and post Five-Times Sit-to-Stand Test

Variables	Pretest Mean ± SD	Posttest Mean ± SD	p value
Heart rate (bpm)	79.7 ± 10.2	86.6 ± 9.7	0.001*
SPO_2_ (%)	96.1 ± 3.0	96.6 ± 3.0	0.348
Systolic blood pressure (mmHg)	118 ± 21.4	129 ± 21.5	0.031*
Diastolic blood pressure (mmHg)	71.3 ± 12.2	75.6 ± 14.2	0.245
Double product (mmHg.bpm)	9,322 ± 1,115.1	11,095 ± 2,804.2	0.114
Borg (PES)	0.52 ± 0.7	1.48 ± 1.4	0.914
Respiratory rate (ipm)	18.7 ± 2.9	20.9 ± 2.7	0.128

SD - standard deviation; SpO_2_ - peripheral oxygen saturation; Borg (PES) - perceived exertion score. T-test for paired samples (p < 0.005).

* Statistically significant.

The only variables that were significantly higher after the test were HR (79.7 ± 10.2bpm/86.6 ± 9.7bpm; p = 0.001) and SBP (118 ± 21.4mmHg/129 ± 21.5mmHg; p = 0.031); however these modifications did not lead to any adverse events.

### Reliability assessment

The mean of the FTSST times for each trial, as well as reliability and standard error of the measurements (SEMs), are presented in table 3. Tests 1 and 3 were performed by the same examiner (test-retest), whereas test 2 was assessed by a different examiner. The mean time of test performance for all trials was 15.30 ± 9.6 seconds.

**Table 3:** Reliability for Five-Times Sit-to-Stand Test for 96 hospitalized patients

Mean trial 1 (SD) [range]	Mean trial 2 (SD) [range]	Mean trial 3 (SD) [range]	ICC_1,2_ [95%CI]	ICC_1,3_ [95%CI]	SEM_1,2_	SEM_1,3_	%SEM_1,2_	%SEM_1,3_
14.97 (9.6)	15.05 (9.5)	14.96 (9.6)	0.99	0.99	0.69	0.68	4.6	4.5
[6.13 - 59.50]	[6.05 - 60.00]	[5.58 - 59.16]	[0.99 - 0.99]	[0.99 - 0.99]				

SD - standard deviation; 95%CI - 95% confidence interval; ICC_1,2_ - intraclass correlation coefficient different examiners; ICC_1,3_ - intraclass correlation coefficient test-retest; SEM_1,2_ - standard error measurement for different examiners; SEM_1,3_ - standard error measurement for test-retest; %SEM%_1,2_ - standard error measurement percentage for different examiners; %SEM_1,3_ - standard error measurement percentage for test-retest.

The means of the FTSST times for each trial were similar, and one-way ANOVA revealed no significant difference between them (p = 0.43). The test-retest reliability (ICC 1,3 = 0.99) and interrater reliability (ICC 1,2 = 0.99) of the FTSST were shown to be excellent. The SEMs for the test-retest and interrater measures were computed to be 0.68 and 0.69 seconds, with SEM percentage change (SEM%) of 4.6% and 4.5%, respectively.

**Figure 2:**
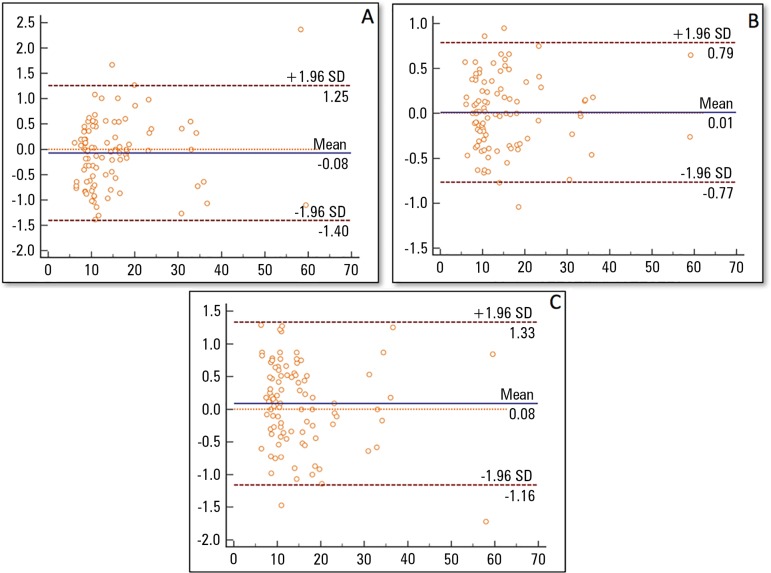
Altman-Bland plot for the five-times sit-to-stand test. The means on the x axis are the average of two trials for the Five-Times Sit-to-Stand Test, and the differences between Five-Times Sit-to-Stand Test scores are in the y axis. (A) Test-retest measurements (Test 1 minus Test 3). (B) Interrater measurements (Test 1 minus Test 2). (C) Interrater measurements (Test 2 minus Test 3). The 95% limits of agreement are depicted (dashed line). SD - standard deviation.

Furthermore, a visual inspection of the Altman-Bland plot (Figure 2) revealed no significant trend towards improving or worsening with regards to test performance. A survival agreement plot analysis showed a degree of agreement of almost 100% between the examiners at values less than 2 seconds, reflecting a very solid degree of agreement.

## DISCUSSION

The purpose of this study was to determine safety, test-retest and interrater reliability of the FTSST in older hospitalized patients being discharged from a general critical care unit to hospital wards. Initially, we observed a low overall rate of patients suitable for the test (29.8%), as well as a low number of patients enrolled in the study (43.8%), considering all patients who met the inclusion criteria. Although current literature information concerning FTSST suitability rates in populations previously investigated is insufficient, a low suitability rate could possibly reflect that the use of FTSST was restricted to a very specific population. Interestingly, 123 patients (57.2%) physically able to perform tests were not enrolled, mainly due to the failure to obtain informed consent for the study. Factors related to refusal were not investigated, however, concern for their current health status and hospitalization could be linked to this finding.

The results showed the test to be safe, as analyzed by hemodynamic and respiratory variable responses (pre- and posttest) and the absence of adverse events. A study published by Suttanon et al.,^(^^21^^)^ also reported the absence of adverse events, including falls in individuals with mild to moderate Alzheimer's disease. Additionally, as seen in table 3, although HR and SBP showed significant statistical increase, it did not lead patients to present an exacerbated response concerning cardiac and respiratory variables. This finding is similar to that found in a study by Morita et al.,^(^^22^^)^ which compared 3 sit-to-stand test (5 reps, 30 seconds and 1 minute) modalities and found that marked changes in SPO_2_, HR, blood pressure, dyspnea and leg fatigue were only found after the 1 minute type.

Regarding test reliability, the results revealed excellent test-retest and interrater reliability, with low percentages of error measurement, as demonstrated by several reliability methods, including ICCs, visual inspection of the Altman-Bland plot and a survival agreement plot. This finding is in accordance with other studies that have investigated FTSST test-retest and interrater reliability.^(^^23,24^^)^ The high interrater and test-retest reliability is possibly related to straightforward test instructions, the researchers' experience and the objective nature of the test assessment as stated by Teo et al.^(^^25^^)^

To date, no data have been referenced in respect to older hospitalized individuals. In a meta-analysis, reference values were established depending on the age group as performing "worse than the average": 11.4 s (60 - 69 years), 12.6 s (70 - 79 years) and 14.8 s (80 - 89 years).^(^^26^^)^ In comparison with these results, in the current research, the mean time of the test was higher (14.98 ± 9.6 s) than for community-based individuals of a similar age group.^(^^22^^)^ Several factors such as chair height, muscle force, use of footwear and trunk, knee and foot position are considered determinants of the sit-to-stand movement and thus influence performance.^(^^27^^)^ Moreover, factors including bed rest during hospitalization, malnutrition, isolation, decrease in muscle mass and other physiologic changes related to bed rest, contributed to overall weakness^(^^28^^)^ and hence, poorer performance. Therefore, original studies are essential to broadly investigate an association between performance during the test, hospitalization process and clinical features.

The present study is a pioneer investigation of FTSST applicability in older Brazilian individuals in a hospital scenario admitted to an intensive care unit. Files et al.,^(^^29^^)^ previously reported that the sit-to-stand test has been administered in the ICU when performing the Short Physical Performance Battery (SPPB). However, there have been no published studies specifically examining the clinimetric properties of either the SPPB test or the FTSST within an ICU setting. The study of the FTSST's clinical applicability through the safety and reliability of measurements is essential to allow a more accurate analysis of functional recovery in older individuals with critical illness.

This test may therefore be helpful as a tool for the risk management of falls and functional decline in hospitalized patients. Innovative studies are necessary to determine its validity and responsiveness in order to establish the fall risk and functional status in this population. Some limitations of the study include the use of a convenience sample and the absence of a power analysis for sample size determination.

## CONCLUSION

Based on the consolidated findings presented, it appears that the Five Times Sit-to-Stand Test is a safe test to be applied to high functioning older individuals at the time of intensive care unit discharge. The Five Times Sit-to-Stand Test presented high interrater and test-retest reliability, and patients recently discharged from intensive care presented a higher score than other previously analyzed populations.
